# Effect of physical training on airway inflammation in bronchial asthma: a systematic review

**DOI:** 10.1186/1471-2466-13-38

**Published:** 2013-06-13

**Authors:** Smita Pakhale, Vanessa Luks, Andrew Burkett, Lucy Turner

**Affiliations:** 1 The Ottawa Hospital, 501 Smyth Road, Ottawa, Ontario K1H 8L6, Canada; 2 Ottawa Hospital Research Institute, Ottawa, Canada; 3 University of Ottawa, Ottawa, Ontario, Canada

**Keywords:** Chronic airway inflammation, Physical exercise, Asthma therapy, Adherence, Meta-analysis

## Abstract

**Background:**

The majority of the global population cannot afford existing asthma pharmacotherapy. Physical training as an airway anti-inflammatory therapy for asthma could potentially be a non-invasive, easily available, affordable, and healthy treatment modality. However, effects of physical training on airway inflammation in asthma are currently inconclusive. The main objective of this review is to summarize the effects of physical training on airway inflammation in asthmatics.

**Methods:**

A peer reviewed search was applied to Medline, Embase, Web of Science, Cochrane, and DARE databases. We included all observational epidemiological research studies and RCTs. Studies evaluating at least one marker of airway inflammation in asthmatics after a period of physical training were selected. Data extraction was performed in a blinded fashion. We decided a priori to avoid pooling of the data in anticipation of heterogeneity of the studies, specifically heterogeneity of airway inflammatory markers studied as outcome measures.

**Results:**

From the initial 2635 studies; 23 studies (16 RCTs and 7 prospective cohort studies) were included. Study sizes were generally small (median sample size = 30). There was a reduction in C-reactive protein, malondialdehyde, nitric oxide, sputum cell counts and IgE in asthmatics with physical training. Mixed results were observed after training for fractional excretion of nitric oxide and bronchial hyperresponsiveness. The data was not pooled owing to significant heterogeneity between studies, and a funnel plot tests for publication bias were not performed because there were less than 10 studies for almost all outcome measures. Physical training intervention type, duration, intensity, frequency, primary outcome measures, methods of assessing outcome measures, and study designs were heterogeneous.

**Conclusion:**

Due to reporting issues, lack of information and heterogeneity there was no definite conclusion; however, some findings suggest physical training may reduce airway inflammation in asthmatics.

## Background

Asthma is a multifactorial disease with genetic, environmental and inflammatory components in its etiology. The principal pathophysiology of asthma is chronic inflammation of the lower respiratory tract [1]. Pharmacotherapy and avoidance of allergens are the primary therapies emphasized in all asthma guidelines [2,3]. Anti-inflammatory agents such as inhaled steroids and leukotriene receptor antagonists along with long acting bronchodilators are the mainstay of asthma pharmacotherapy; however, persistent inflammatory cell infiltration has been demonstrated even after a course of oral steroids (methyl-prednisone 40 mg daily for 14 days) [4]. In addition, airway remodeling in established asthma responds poorly to current medicinal therapies [5]. Current asthma therapies have not achieved asthma prevention or asthma cure and there are currently no medications that can alter the natural history of the disease [6]. Potential long term side-effects, prohibitive costs, and suboptimal adherence to asthma medications are on-going challenges to optimal asthma control. Even for the newer and expensive asthma therapies, such as omalizumab, only a very small subpopulation of refractory or severe asthma appears to respond [7,8]. Treatment options are therefore quite limited for asthma and the need to search for other therapies has been recognized by many experts in the field [4,5,9].

Physical exercise training is thought to be beneficial in asthma management, at least in children, but it has not been extensively studied [10]. There are no specific recommendations on physical training type, intensity, duration, or frequency in any asthma guidelines [2,3]. Moreover, low physical activity in asthmatics is a reality because they usually avoid exercise [11]. Asthma and chronic obstructive pulmonary disease (COPD) are, according to the ‘Dutch hypothesis’ [12,13], different manifestations of the same disease entity. In addition, asthma and COPD might share common pathogenetic pathways [14]. Positive impacts of physical exercise training and rehabilitation in COPD have been extensively studied [15,16]; therefore, physical training is recommended in COPD guidelines [15,17,18]. Given that asthma and COPD may share a common pathogenesis, and that both diseases are manifested by chronic airway inflammation, it is imperative that we discern the role of physical training in asthma management. This systematic review represents an endeavour to shed light on this relationship, as we assess the effects of physical training on markers of airway inflammation in asthmatics.

To the best of our knowledge, there exists no systematic review to date focusing on the effects of physical training on airway inflammation in asthmatics. A Cochrane review published in 2012 summarized the effects of physical training on asthma and included only randomized controlled trials (RCTs) [10]. The objective of this review was to extend the evidence base by using all types of observational epidemiologic research in combination with RCTs [19] to summarize effects of physical training on airway inflammation in asthmatics.

## Methods

A separate protocol for this review was not previously published.

### Data sources and search

Studies were identified by searching electronic databases, and scanning reference lists of articles. The search was applied to Medline (1948 – 2012), Embase (1947 – 2012), and adapted for Web of Science (1898 – 2012) including SCI-EX (Science citation index Expanded), SCPCI-S (Conference Proceedings Citation Index – Science), and SPCI-SSH (Conference Proceedings Citation Index). It was also adopted for Cochrane (CENTRAL), and DARE (Database of Abstract of Reviews of Effectiveness), and the Cochrane Database of Systematic Reviews (Cochrane Reviews). These databases were searched for published studies that included physical training program in asthmatics. Variants of key words such as “Physical Education and Training”, “physical activity”, “physical training” were used. The search was run on April 19th, 2012. As we planned to investigate both humans and animal models, the search was not restricted to human subjects. No language or region restrictions were applied. The search was peer-reviewed by a librarian at The Ottawa Hospital, Ottawa, Canada.

### Full electronic search strategy

The following search strategy was applied to Medline and Embase (peer-reviewed by a librarian at The Ottawa Hospital, Ottawa, Canada):

(exp Asthma/ OR asthma.tw) AND (exp Exercise/ or exp Exercise Therapy/ or exp Exercise Movement Techniques/ or exp Physical Conditioning, Animal/ or exp "Physical Education and Training"/ or exp Physical Exertion/ or (exercis$ or aerobic train$ or physical activi$).tw) AND (inflammation or inflammatory).tw or Inflammation/ or exp Inflammation Mediators/or bronchial biopsy.mp or Cell Count/ or Sputum/ or Adenosine Monophosphate/ or Methacholine Chloride/ or Histamine/ or tumstatin.mp or Basement Membrane/ or Collagen/ or Collagen Type IV/ or Immunoglobulin E/ or T-Lymphocytes, Regulatory/ or eicosanoids/ or leukotrienes/ or prostaglandins/ or thromboxanes/ or cytokines/ or chemokines/ or interleukin-8/ or platelet factor 4/ or interferons/ or interferon type i/ or interferon-gamma/ or interleukins/ or interleukin-1/ or interleukin-2/ or interleukin-3/ or interleukin-4/ or interleukin-5/ or interleukin-6/ or interleukin-7/ or interleukin-9/ or interleukin-11/ or interleukin-12/ or interleukin-13/ or interleukin-15/ or interleukin-16/ or interleukin-17/ or interleukin-18/ or lymphokines/ or leukocyte migration-inhibitory factors/ or macrophage-activating factors/ or transforming growth factor beta/ or tumor necrosis factors/ or tumor necrosis factor-alpha/ or endothelial growth factors/ or fibroblast growth factors/ or platelet-derived growth factor/ or tolloid-like metalloproteinases/ or transforming growth factors/ or cells/ or antigen-presenting cells/ or granulocytes/ or basophils/ or eosinophils/ or neutrophils/ or leukocytes, mononuclear/ or cytokine-induced killer cells/ or killer cells, lymphokine-activated/ or monocytes, activated killer/ or t-lymphocytes, cytotoxic/ or lymphocytes/ or killer cells, natural/ or lymphocyte subsets/ or t-lymphocyte subsets/ or t-lymphocytes/ or cd4-positive t-lymphocytes/ or cd8-positive t-lymphocytes/ or natural killer t-cells/ or monocytes/ or fibroblasts/ or mast cells/ or epithelial cells/ or goblet cells/ or phagocytes/ or histiocytes/ or Nitric Oxide/ or lung inflammation.mp or Th2 Cells/ or Bronchoalveolar Lavage Fluid/ or airway inflammation.mp. or Bronchial Hyperreactivity/).

### Study selection

A scoping search revealed few studies in this area pertaining to either humans or animal models of asthma; therefore, we decided to investigate this question for both animal models and human asthmatic subjects of all age groups (the former published separately [20]). For this review, the inclusion criteria were 1) subjects with asthma as per the Canadian Thoracic Society Asthma Guidelines [3], or the Global Initiative for Asthma (GINA) guidelines, or physician diagnosed asthma [2]); 2) the intervention - a physical training program; and 3) at least one marker of airway inflammation evaluated at the end of the training program. Importantly, we included all observational epidemiological research studies and RCTs. In addition, we also included studies satisfying inclusion criteria published in abstract forms only. We generated a list of candidate markers of airway inflammation based on literature searches, but allowed markers not on this list if there was appropriate justification.

### Data extraction and quality assessment

The search was applied to each database and the results were combined. Duplicate articles were removed. For the first search stage, two authors (V.L. and A.B.) independently scanned the titles and abstracts for selecting studies. At the first stage, studies were included if insufficient information was available to reliably exclude them. For the second stage, the full text of each study was obtained, and both authors independently applied the inclusion and exclusion criteria blinded to author and publication data. Studies meeting the inclusion/exclusion criteria were included for data extraction. Finally, the reference lists of all studies from stage 2 were reviewed to ensure that all relevant studies were considered. If there were any disagreements as to whether an article should be included or excluded, a third reviewer (S.P.) was called upon to reach a consensus. Human studies were used for this review, and animal asthma model studies were set aside for a separate review [20].

Data extraction forms were created and piloted on six articles obtained from the initial scoping search. The data extraction forms were then modified to facilitate extraction of the relevant data. The data was extracted independently by two authors (V.L. and A.B.) blinded to the study authors, and publication date. Any disagreements were resolved by consensus after reviewing the study. The accuracy of the information was not verified with the authors of the original studies. Studies where the data was represented in graphical format only, or if there was insufficient raw numerical data to perform meta-analysis, study authors were contacted [21-25]; we successfully obtained further information only for three of these studies [21-23].

The following data was extracted from each of the included studies if available: study design, characteristics of subjects (including method of diagnosis of asthma, baseline asthma severity, baseline medications, atopy, body mass index, smoking status, baseline exercise routine), physical training intervention/s (type of training, intensity and duration of each session, frequency of sessions, duration of training program), control intervention (if any), list of markers of airway inflammation, and the change in the evaluated markers of airway inflammation with physical training.

To determine the validity of the included trials one author (A.B.) applied the Cochrane risk of bias tool to RCTs [26] and the Ottawa Newcastle tool for all observational epidemiologic research studies to assess study-level and outcome-level risk of bias [27].

### Data synthesis and analysis

We anticipated heterogeneity in studies in the primary outcome measures, methods of assessing outcome measures, and study designs. It is difficult to interpret pooled or summary measures in the presence of significant heterogeneity [28]. We decided a priori to avoid pooling of the data in anticipation of heterogeneity of the studies, specifically heterogeneity of airway inflammatory markers studied as outcome measures. Hence, no pooled estimates of effect were calculated.

We grouped the studies measuring similar markers of airway inflammation as outcome measures creating forrest plots for their mean differences to aid visual interpretation of effect estimates. We converted medians to means where the data appeared normally distributed in the graphs. Using appropriate formulae, we converted interquartile ranges, measures of variation, and 95% confidence intervals to standard deviations where the data appeared normally distributed [28].

Though many studies reported on exercise-induced bronchoconstriction, this outcome was measured in a myriad of different ways hence the results were described only qualitatively.

It is unknown if there was any selective reporting bias, as protocols for individual studies were not widely available. Funnel plots were not performed because, except EIB, there were less than ten studies reporting on any single outcome [29]. Though there were 13 studies reporting on EIB, data was heterogeneous.

Data analysis was performed using the software Comprehensive Meta-Analysis version 2.0. [30].

## Results

### Search

**Figure 1:**
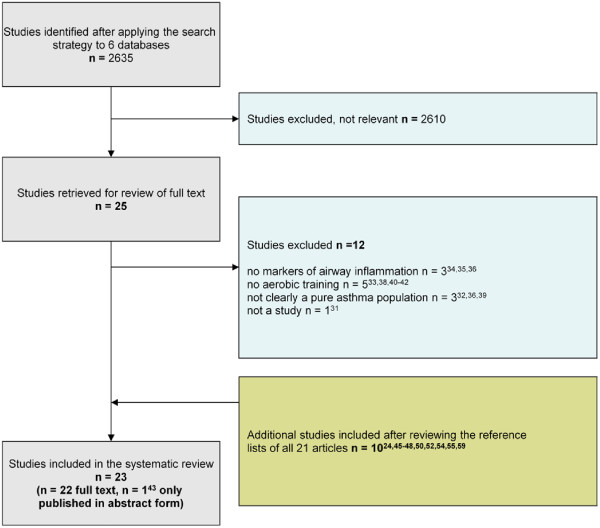
Flow chart of systematic search.

The initial search yielded a total of 2635 citations. Twenty-five potentially eligible studies were selected in stage 1 based on titles and abstracts; inter-reviewer agreement was 100%. In stage 2, full texts of these 25 articles were reviewed; twelve studies were excluded for reasons stated in Figure 1 (100% inter-reviewer agreement) [31-42]. After searching the reference lists of all articles from stage 2, an additional 10 studies met inclusion criteria. Therefore, the total number of studies in the final analysis were 23 (22 available in full text, 1 published only in abstract form [43]. We do not think there is significant publication bias as there are a number of published studies with negative results. However, we could not draw a funnel plot to evaluate this assumption graphically, because there were less than 10 studies for almost all outcome measures [29].

### Studies

Additional file 1 summarizes the important characteristics of the included studies. There were 16 randomized controlled trials [21,25,43-56] and 7 prospective cohort studies [22-24,57-60]. Diagnosis of asthma was not uniform in all the studies with many studies relying solely on physician diagnosis of asthma or did not provide explicit details of method of diagnosis. There were 5 studies with mild asthma (2 with controlled [44,51] and 3 with unknown control status [53,55,58], 9 studies with mild to moderate asthma (3 with controlled [21,23,46] and 6 with unknown control status [22,25,43,50,57,60], 6 studies with moderate to severe asthma (1 with controlled [47] and 5 with unknown control status (24;45;52;54;56)), 1 study with severe asthma with unknown control status [59] and 2 studies did not clearly mention asthma severity or control [48,49]. The median sample size in the included studies was 30 (interquartile range (IQR) 1–3: 6–171) and the median age of asthmatic subjects was 10 years (IQR1-3: 5–70). The studies included median of 43% females (IQR1-3: 0–82). Six studies did not report sex of asthmatic subjects and one study did not report ages of participating children. Study subjects underwent physical training either on land or water for a median duration of 45 minutes (IQR1-3: 30–120 minutes) per session. One study had a competitive running program of 3.2 km and another study had walking at 60-75% of age-predicted maximum heart rate. Median frequency of physical training was 2 times per week (IQR1-3: 0.2-6) for a median total duration of 12 weeks (IQR1-3: 2–156).

### Risk of bias

We determined study-level and outcome-level risk of bias (Tables 1 and 2). Eight RCTs had low risk of bias due to randomization but eight had unclear risk as explicit details about randomization were not reported. Ten studies had sufficient concealment of allocation to protect against bias but six had unclear risk. While the blinding of participants in studies with physical training as an intervention is impossible, it is possible to perform outcome assessments in a blinded fashion. Importantly, most studies did perform outcome assessments in blinded fashion. Incomplete outcome data was not an issue in any included studies however selective reporting bias could not be commented due to unavailability of protocols. Selection, information, and confounding bias posed a low risk in all included cohort studies. Though appropriate analytic strategies were used in all included cohort studies, risk due to appropriate sample size was unclear. Additional file 2 enlists the PRISMA Checklist.

**Table 1:** Risk of bias within randomized control trials

**Source**	**Randomization**	**Allocation concealment**	**Blinding of participants & personnel**	**Blinding of outcome assessment**	**Incomplete outcome data**	**Selective reporting**
Bonsignore MR et al. 2008	Low risk	Unclear risk	Low risk	Low risk	Low risk	Unclear risk
Boyd, A. et al. 2011	Low risk	Unclear risk	Low risk	Low risk	Low risk	Unclear risk
Bundgaard A, et al. 1982	Unclear risk	Low risk	Low risk	Low risk	Low risk	Unclear risk
Cochrane LM et al.	Low risk	Low risk	Low risk	Low risk	Low risk	Unclear risk
Emtner, M. et al. 1998	Low risk	Unclear risk	Low risk	Low risk	Low risk	Unclear risk
Fanelli A, et al. 2007	Low risk	Low risk	Low risk	Low risk	Low risk	Unclear risk
Fitch, KD et all, 1986	Unclear risk	Unclear risk	Low risk	Low risk	Low risk	Unclear risk
Gunay, O. et al. 2012	Unclear risk	Low risk	Low risk	Low risk	Low risk	Unclear risk
Henriksen JM, et al. 1983	Unclear risk	Low risk	Low risk	Low risk	Low risk	Unclear risk
Matsumoto I, et al. 1997	Low risk	Unclear risk	Low risk	Low risk	Low risk	Unclear risk
Mendes, et al. 2011	Low risk	Low risk	Low risk	Low risk	Low risk	Unclear risk
Moreira, A et al. 2008	Low risk	Low risk	Low risk	Low risk	Low risk	Unclear risk
Neder JA, et al. 1999	Unclear risk	Low risk	Low risk	Low risk	Low risk	Unclear risk
Onur E, et al. 2011	Unclear risk	Low risk	Low risk	Low risk	Low risk	Unclear risk
Sly RM, et al. 1972	Unclear risk	Unclear risk	Low risk	Low risk	Low risk	Unclear risk
Svenonius E, et al. 1983	Unclear risk	Low risk	Low risk	Low risk	Low risk	Unclear risk

**Table 2:** Risk of bias within cohort studies

**Source**	**Selection bias**	**Information bias**	**Confounding**	**Appropriate sample size**	**Appropriate analytic strategies**
Emtner M et al. 1998	Low risk	Low risk	Low risk	Unclear risk	Appropriate
Emtner M et al. 1996	Low risk	Low risk	Low risk	Unclear risk	Appropriate
Engstom I, et al. 1991	Unclear risk	Low risk	Low risk	Unclear risk	Appropriate
Juvonen R, et al. 2008	Low risk	Low risk	Low risk	Unclear risk	Appropriate
Newcomb, P. et al. 2012	Unclear risk	Low risk	Low risk	Unclear risk	Appropriate
Nickerson BG et al., 1983	Low risk	Low risk	Low risk	Unclear risk	Appropriate
Silva PL, et al. 2011	Low risk	Low risk	Low risk	Low risk	Appropriate

### Results of individual studies

#### Yes, physical exercise may reduce airway inflammation

**Multiple studies with same outcome measure** C - reactive protein (CRP)

Three studies reported serum C-reactive protein (CRP) (51;57;60). All three studies demonstrated significantly decreased CRP mean estimates with physical exercise. Manual conversion of graphically presented data in two studies (51;60) did not accurately reflect the original findings and hence, we chose not to depict the CRP data graphically in this paper.

#### Single studies

A single study reported on the reactive oxygen species malondialdehyde (MDA) and total plasma nitric oxide (NO), as well as the antioxidants serum superoxide dismutase (SOD), and glutathione peroxidase (GHS-Px) [53]. The NO in the asthmatic groups decreased with exercise and inhaled corticosteroids (ICS), beyond the effects of ICS alone; and furthermore, NO levels returned to control (non-asthmatic) levels. The addition of exercise to ICS in the asthmatic group did not increase SOD or MDA beyond the effects of ICS alone. Lastly, glutathione peroxidase was not increased by ICS alone in asthmatics, but did increase with the combination of ICS and exercise to levels exceeding those in the non-asthmatic control group.

A single study reported on sputum cell counts and found decreased total cells and eosinophils in asthmatics post-training, but there was no change in sputum macrophages, neutrophils, lymphocytes, epithelial cells, squamous cells, goblet cells, or ciliated cells [56].

One study reported a decrease in serum IgE and in mite-specific IgE in the physically trained group. However, it must be noted that, the study was performed from February to March and there was also a significant decrease in these values in the control group. This raises the question of whether the IgE reduction was due to the subjects spending more time outside with less exposure to dust mites [51].

Gunay et al. studied serum endothelin-1, matrix metalloproteinase-9, MDA and urine leukotriene E4 (LTE4) in asthmatics on pharmacologic therapy in comparison to those on pharmacologic therapy and exercise training. Serum endothelin-1 was decreased in the group allocated to pharmacologic therapy and exercise only; however, the urine LTE4, MDA and serum matrix metalloproteinase-9 decreased to the same extent in both the groups [49].

A single study reported on adiponectin and leptin in asthmatics after one year of training [60]. Adiponectin an anti-inflammatory protein hormone was increased and leptin a pro-inflammtory protein hormone was decreased post-training.

### Mixed results, physical exercise may not reduce airway inflammation

#### Multiple studies with same outcome measure

**Figure 2:**
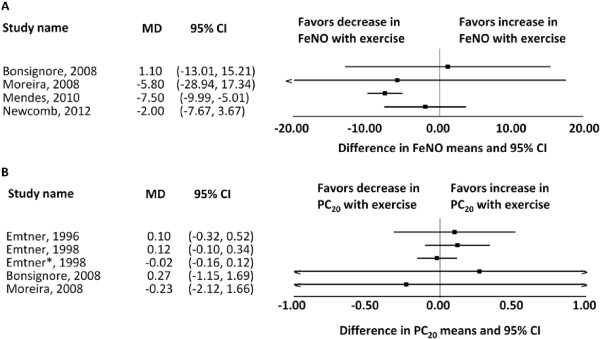
**Forest plots of results. A**. Difference in means of FeNO in asthmatics pre- and post-exercise training. **B**. Difference in means of PC20 for methacholine in asthmatics pre- and post-exercise training. (Emtner* 1998 (23) is a 3 year follow-up study of the patients from the Emtner 1996(22) and Emtner 1998(21) studies.).

**Fractional excretion of nitric oxide (FeNO)** Four studies reported fractional excretion of nitric oxide (FeNO) shown in Figure 2A. Three studies showed no difference after training [44,51,58], and one study reported decreased FeNO in the trained group compared to baseline and compared to the control group [56].

Bronchial hyperresponsiveness to methacholine or histamine

Eight studies reported on bronchial hyperresponsiveness to either methacholine or histamine [21-25,44,46,51]. Six of these studies showed no change in the non-specific bronchial hyperreactivity to either agent post-training [21-23,25,46,51]. Of note, one of these studies [23] is a three year follow-up of the patients in two other studies [21,22]. Engstrom et al. reported 50% of subjects having an increase in tolerance to histamine of more than one dose step; however, there was no control group and the study number was very small [24]. Bonsignore et al. reported a significantly increased PD20 post- versus pre-training in a group of subjects randomized to exercise (p < 0.02). Of note, examining the plotted raw data, it appears not to be significant (Figure 2B); however, this is likely due to converting the median and range reported in the study into a mean and standard deviation [44]. The studies for which we could reliably extract numerical data are graphically represented above (Figure 2B).

##### 

**Exercise-induced bronchoconstriction (EIB)** The effect of training on exercise-induced bronchoconstriction was reported in thirteen studies (21–23;25;44;45;47;48;50;52;54;55;59). The criteria for exercise-induced bronchoconstriction varied between studies with some relying on peak expiratory flow analysis, and the exercise test protocols used to measure EIB also varied. Eight of these studies showed decreased EIB post-training but many of these results are marred by serious methodological flaws including relatively reduced ventilator stress at the post-training test owing to improvements in fitness during the training period, possible use of β2 agonists pre-testing, and the possible role of medication changes during the training period that could have contributed to a reduction in EIB (21–23;25;44;47;50;55).

##### Single studies

Only one study reported on serum eosinophils and there was no difference between controls and the trained group [51]. The same study also reported on eosinophilic cationic protein and found no difference after training [51].

One study reported on exhaled breath condensate of cysteinly leukotrienes and found no change post training [44].

## Discussion

Physical exercise has multi-faceted benefits in health and disease [61,62] and is therefore part of many guidelines for chronic diseases [63]. Physical exercise is a suggested strategy for targeting T-cells in diseases like asthma [64], and has been shown to reduce airway inflammation in animal models of asthma [20]. It remains unclear, however, whether physical exercise reduces airway inflammation in human asthmatics. Specific recommendations on physical exercise dose, duration and frequency hence are not included in current asthma guidelines [2,3].

Physical exercise does seem to have beneficial effects on reducing airway inflammation as shown by reduction in CRP [51,57,60]. CRP is a systemic inflammatory marker, used often for disease monitoring during treatment. In asthmatic subjects, in absence of any other systemic inflammatory disease, reduction in CRP could presumably be due only to a reduction in airway inflammation.

The results from single studies do demonstrate that there is a signal for reduction in airway inflammation in response to physical training; however, we cannot conclude the benefits based upon the data from single studies. Nevertheless, the effects on reactive oxygen species after physical training are important: there is reduction in malondialdehyde (MDA) and total plasma nitric oxide (NO), no further increase in SOD beyond that seen with ICS alone, and increase in GSH-Px with the combination of ICS and exercise [53]. Similarly, the finding of decreased total sputum cells and eosinophils in asthmatics post-training [56], decreased serum endothelin-1 [49], and increased adiponectin and decreased leptin post-training [60] are significant and could be due to reduction in airway inflammation. Adiponectin and leptin however could represent an increased systemic inflammatory milieu from obesity [65,66]. An increase in the prevalence of asthma and obesity has been linked [67-69], however the directionality of this relationship is unclear; it is yet unknown whether the presence of asthma leads to obesity or whether obesity leads to development of asthma.

Multiple studies have evaluated fractional excretion of Nitric Oxide (FeNO) (Figure 2A), bronchial hyperresponsiveness to methacholine or histamine (Figure 2B) and exercise-induced bronchoconstriction (EIB) with mixed results. Noninvasive inflammatory markers in asthma such as FeNO demonstrated heterogeneous results. As such, the literature remains unconvincing and inconclusive on the role of FeNO in asthma management, as FeNO could be affected by non-disease related factors such as atopy, height/age and infection [70,71]. Though there were eight studies estimating bronchial hyperresponsiveness, we could not combine the results in a forest plot as bronchial hyperresponsiveness was measured to either methacholine or histamine. Six of these eight studies did not show any significant change which could be due to the non-specific nature of the agent provoking bronchial hyperreactivity. Bonsignore et al. demonstrated that airway response to methacholine and EIB decreased after 12 weeks of training with or without montelukast [44]. Improvements in EIB, if they were real and not related to a decreased ventilator load post training, suggest that the mechanism of provocation in exercise is different compared to methacholine [22,23,44] and that the former can be positively impacted by training. These results are not conclusive because methods used in these studies varied significantly and were not standardized.

Finally, the method of diagnosis of asthma was not uniform in all the studies which could introduce selection bias (please see the Additional file 1). Establishing a diagnosis of asthma can be challenging even at a tertiary care center because of its variable natural history. Even though systematic, algorithmic diagnosis of asthma is cost-effective, asthma misdiagnosis could be as common as 30% [72-74]. Asthma misdiagnosis in the studies included in this review might drive airway inflammation outcome data towards null if some truly non-asthmatic subjects were misclassified as asthmatics; and hence might nullify effects of physical training.

This systematic review and the Cochrane Review [10] have demonstrated the main problem: there are very few RCTs and observational studies that have evaluated the effects of physical exercise training on asthmatic subjects and all are plagued by small sample sizes (10;20). One non-randomized controlled study [75] with 15 adult asthmatics demonstrated a clinically significant improvement in Asthma Control (0.5 increase) as measured by the Asthma Control Questionnaire (ACQ) and improvements in quality of life, as measured by the Asthma Quality of Life Questionnaire (AQLQ) with the structured-physical exercise program. The US Nurses’ Health Study cohort database demonstrated prospectively in 2818 asthmatics that physical exercise (median physical activity = walking at a brisk pace for 20 minutes three times per week) might be associated with lower risk of asthma exacerbation irrespective of asthma severity and other covariates [76]. This database study demonstrated that there was a dose–response relationship; higher the level of physical activity lower the risk of asthma exacerbation [76].

This systematic review provides important insight into the role of physical training in the treatment of asthma in children and adults. There is some evidence that physical training might provide beneficial effects by reducing airway inflammation in asthmatics, though this cannot be conclusively proven from the existing literature. Physical training seems to be safe in asthmatics [10] though we do not know the safety limits of duration, intensity, and frequency.

### Strengths and limitations of the review

The current review has several strengths. We have employed comprehensive search strategies using multiple databases of published literature and employed standardized methods of conducting systematic reviews. Despite our comprehensive efforts, it is possible that we have missed some relevant studies in the process of publication or other unpublished sources.

Heterogeneity of the included studies was predicted and confirmed and hence data was not pooled. We described the data, when possible, using forest plots. Many studies did not use standardized methods of testing (e.g. testing protocols for EIB), thereby limiting the conclusions that could be drawn. In addition, reporting was not uniform across studies, especially e.g. asthma diagnosis, asthma medications, asthma severity and control. Finally, different markers of airway inflammation were used in different studies, leading to few studies reporting on any single outcome. Most importantly, physical training intervention(s) (type of training, intensity and duration of each session, frequency of sessions, duration and site of training program) and control intervention(s) were heterogeneous across studies. These issues combined to limit the strength of our conclusions.

## Conclusions

Effectiveness of physical exercise on airway inflammation is yet unproven. There is, however, some evidence to suggest that physical exercise may reduce inflammation in an asthmatic airway. Should such evidence emerge in future research, physical exercise may well prove to be an affordable, accessible, healthy, non-invasive, and enjoyable asthma management strategy. We urgently need to design studies to understand if this non-pharmacological adjunctive strategy can effectively decrease airway inflammation, control symptoms of asthma, and be safely administered in the community.

## Competing interests

No competing interests for any author.

## Authors’ contributions

SP had full access to the data and takes full responsibility for the integrity of the data and the accuracy of the data analysis, contributed to the concept, design, implementation, statistical analysis, interpretation and writing. VL contributed to the data management, statistical analysis, interpretation and writing. AB contributed to the data management, statistical analysis, interpretation and writing. LT contributed to the data analysis, interpretation and writing. All authors read and approved the final manuscript.

## Pre-publication history

The pre-publication history for this paper can be accessed here:

http://www.biomedcentral.com/1471-2466/13/38/prepub

## Supplementary Material

Additional file 1 **Summary of included studies.** This file provides details of the studies included in this systematic review including the number of subjects, mode of asthma diagnosis, asthma severity and control, current asthma pharmacotherapy, study design, training intervention (dose, duration, site, intensity and frequency of physical exercise), control intervention and outcome measures. Click here for file

Additional file 2 PRISMA Checklist. Click here for file

## References

[B1] Hargreave FE Nair P The definition and diagnosis of Asthma Clin Exp Allergy 2009 39 11 1652 1658 10.1111/j.1365-2222.2009.03321.x 19622089

[B2] Global Initiative for Asthma Report, Global Strategy for Asthma Management and Prevention: Updates December 2011 2002 Accessed on 2012 June 25. Available from: http://www.ginasthma.org/uploads/users/files/GINA_Report2011_May4.pdf

[B3] Lougheed MD Lemiere C Ducharme FM Licskai C Dell SD Rowe BH Canadian Thoracic Society 2012 guideline update: diagnosis and management of asthma in preschoolers, children and adults Can Respir J 2012 19 2 127 164 22536582 10.1155/2012/635624 PMC3373283

[B4] Chakir J Hamid Q Bosse M Boulet LP Laviolette M Bronchial inflammation in corticosteroid-sensitive and corticosteroid-resistant asthma at baseline and on oral corticosteroid treatment Clin Exp Allergy 2002 32 4 578 582 10.1046/j.0954-7894.2002.01323.x 11972605

[B5] Sumi Y Hamid Q Airway remodeling in asthma Allergol Int 2007 56 4 341 348 10.2332/allergolint.R-07-153 17965577

[B6] Editorial Asthma: still more questions than answers Lancet 2008 372 9643 1009 10.1016/S0140-6736(08)61414-2 18805308

[B7] Pakhale S Mulpuru S Boyd M Optimal management of severe/refractory asthma Clinic Med Insights Circ Respir Pulmonary Med 2011 5 CMCRPM-5-5535-Pakhale 37 10.4137/CCRPM.S5535 PMC3165919 21912491

[B8] Holgate S Pathophysiology of asthma: what has our current understanding taught us about new therapeutic approaches? J Allergy Clin Immunol 2011 128 3 495 505 10.1016/j.jaci.2011.06.052 21807404

[B9] Holgate ST Has the time come to rethink the pathogenesis of asthma? [Miscellaneous] Curr Opin Allergy Clin Immunol 2010 10 1 48 53 10.1097/ACI.0b013e3283347be5 19915457

[B10] Chandratilleke MG Carson KV Picot J Brinn MP Esterman AJ Smith BJ Physical training for asthma Cochrane Database of Systematic Reviews 2012 5 Art.No.: CD001116 10.1002/14651858.CD001116.pub3 22592674

[B11] Williams B Powell A Hoskins G Neville R Exploring and explaining low participation in physical activity among children and young people with asthma: a review BMC Fam Pract 2008 9 1 40 10.1186/1471-2296-9-40 18590558 PMC2447841

[B12] Orie NGM Slutter HJ De V Tammeling GJ Chronic nonspecific respiratory diseases Ned Tijdschr Geneeskd 1961 105 2136 2139 1961;105:2136–9 14482224

[B13] Van Eerdewegh P Little RD Dupuis J Del Mastro RG Falls K Simon J Association of the ADAM33 gene with asthma and bronchial hyperresponsiveness Nature 2002 418 6896 426 430 10.1038/nature00878 12110844

[B14] Hunninghake GM Cho MH Tesfaigzi Y Soto-Quiros ME Avila L Lasky-Su J MMP12, lung function, and COPD in high-risk populations N Engl J Med 2009 361 2599 2608 10.1056/NEJMoa0904006 20018959 PMC2904064

[B15] O'Donnell DE Hernandez P Kaplan A Aaron S Bourbeau J Marciniuk D Canadian Thoracic Society recommendations for management of chronic obstructive pulmonary disease - 2008 update - highlights for primary care Can Respir J 2008 15 1A 8A 18292855 10.1155/2008/641965 PMC2802325

[B16] Maltais F Bourbeau J Shapiro S Lacasse Y Perrault H Baltzan M Effects of home-based pulmonary rehabilitation in patients with chronic obstructive pulmonary disease Ann Intern Med 2008 149 12 869 878 10.7326/0003-4819-149-12-200812160-00006 19075206

[B17] Lacasse YF Goldstein RF Lasserson TJ FAU Martin S Pulmonary rehabilitation for chronic obstructive pulmonary disease Cochrane Database of Systematic Reviews 2006 4 Art. No.: CD003793 10.1002/14651858.CD003793.pub2 17054186

[B18] Marciniuk DD Brooks D Butcher S Debigare R Dechman G Ford G Optimizing pulmonary rehabilitation in chronic obstructive pulmonary disease–practical issues: a Canadian Thoracic Society Clinical Practice Guideline Can Respir J 2010 17 4 159 168 20808973 10.1155/2010/425975 PMC2933771

[B19] Dreyer NA Making observational studies count: shaping the future of comparative effectiveness research Epidemiology 2011 22 3 295 297 10.1097/EDE.0b013e3182126569 21464648

[B20] Luks V Burkett A Turner L Pakhale S Effect of physical training on airway inflammation in animal models of asthma: a systematic review BMC Pulm Med 2013 13 1 24 10.1186/1471-2466-13-24 23617952 PMC3691924

[B21] Emtner M Finne M Stalenheim G High-intensity physical training in adults with asthma. A comparison between training on land and in water Scand J Rehabil Med 1998 30 4 201 209 10.1080/003655098443940 9825384

[B22] Emtner M Herala M St + Nlenheim G High-intensity physical training in adults with asthma Chest 1996 109 2 323 330 10.1378/chest.109.2.323 8620700

[B23] Emtner M Finne M Stσlenheim G A 3-year follow-up of asthmatic patients participating in a 10-week rehabilitation program with emphasis on physical training Arch Phys Med Rehabil 1998 79 5 539 544 10.1016/S0003-9993(98)90070-3 9596396

[B24] Engstrom I Fallstrom K Karlberg E Sten G Bjure J Psychological and respiratory physiological effects of a physical exercise programme on boys with severe asthma Acta Paediatr Scan 1991 80 1058 1065 10.1111/j.1651-2227.1991.tb11783.x 1750339

[B25] Matsumoto I Araki H Tsuda K Odajima H Nishima S Higaki Y Effects of swimming training on aerobic capacity and exercise induced bronchoconstriction in children with bronchial asthma Thorax 1999 54 3 196 201 10.1136/thx.54.3.196 10325893 PMC1745437

[B26] Higgins JPT Altman DG Sterne JAC Chapter 8: Assessing risk of bias in included studies Cochrane Handbook for Systematic Review of Interventions Cochrane handbook for systematic review of interventions. The Cochrane Collaboration 2008

[B27] Wells GA Shea B O'Connell D Peterson J Welch V Losos M The Newcastle-Ottawa Scale (NOS) for assessing the quality of nonrandomised studies in meta-analyses Accessed on 2012 June 25. Available from: URL: http://www.ohri.ca/programs/clinical_epidemiology/oxford.asp

[B28] Deeks JJ Higgins JPT Altman DG Higgins JPT, Green S Chapter 9: Analysing data and undertaking meta-analyses Cochrane Handbook for Systematic Review of Interventions Cochrane handbook for systematic review of interventions. The Cochrane Collaboration 2008

[B29] Sterne JAC Sutton AJ Ioannidis JPA Terrin N Jones DR Lau J Recommendations for examining and interpreting funnel plot asymmetry in meta-analyses of randomised controlled trials BMJ 2011 342 1 8 10.1136/bmj.d4002 21784880

[B30] Borenstein M Hedges L Higgins J Rothstein H Comprehensive Meta-analysis 2005 http://www.meta-analysis.com/ Version 2, Biostat, Englewood NJ

[B31] Sport is beneficial for individuals with exercise-induced asthma Sport is beneficial for individuals with exercise-induced asthma Drugs Ther Perspect 1998 11 4 5 7 10.2165/00042310-199811040-00002

[B32] Bijl D Speelberg B Folgering HT Pulmonary rehabilitation at moderate altitude: a 1-year follow-up Neth J Med 1994 45 4 154 161 7808577

[B33] Bougault V Turmel J St-Laurant J Bertrand M Boulet LP Asthma, airway inflammation and epithelial damage in swimmers and cold-air athletes Eur Respir J 2009 33 4 740 746 10.1183/09031936.00117708 19129276

[B34] Durigan JLQ Peviani SM Russo TL Duarte AC Vieira RP Martins MA Physical training leads to remodeling of diaphragm muscle in asthma model Int J Sports Med 2009 30 6 430 434 10.1055/s-0028-1112145 19199218

[B35] Durigan JLQ Peviani SM Russo TL Silva ACD Vieira RP Martins MA Effects of exercise training on atrophy gene expression in skeletal muscle of mice with chronic allergic lung inflammation Braz J Med Biol Res 2009 42 4 339 345 19330261 10.1590/s0100-879x2009000400005

[B36] Helenius I Rytila P Sarna S Lumme A Helenius M Remes V Effect of continuing or finishing high-level sports on airway inflammation, bronchial hyperresponsiveness, and asthma: a 5-year prospective follow-up study of 42 highly trained swimmers J Allergy Clin Immunol 2002 109 6 962 968 12063525 10.1067/mai.2002.124769a

[B37] Holzer FJ Schnall R Landau L The effect of a home exercise programme in children with cystic fibrosis and asthma J Paediatr Child Health 1984 20 4 297 302 10.1111/j.1440-1754.1984.tb00098.x 6529386

[B38] Koul PA Effects of exposure to cold and exercise on bronchial asthma Drugs Ther Perspec 1998 11 4 5 7 10.2165/00042310-199811040-00002

[B39] Nystad W Stigum H Carlsen KH Increased level of bronchial responsiveness in inactive children with asthma Respir Med 2001 95 10 806 810 10.1053/rmed.2001.1149 11601746

[B40] Vempati R Deepak K Effect of yogic practices on airway inflammation, mast cell activation and exercise induced asthma: a randomized controlled trial Allergy 2007 62 217

[B41] Vempati R Bijlani RL Deepak KK The efficacy of a comprehensive lifestyle modification programme based on yoga in the management of bronchial asthma: a randomized controlled trial BMC Pulm Med 2009 9 37 10.1186/1471-2466-9-37 PMC2734746 19643002

[B42] Scott HA Gibson PG Garg ML Wood LG Airway inflammation is augmented by obesity and fatty acids in asthma Eur Respir J 2011 38 3 594 602 10.1183/09031936.00139810 21310876

[B43] Boyd AW Estell K Dransfield M Schwiebert L The effect of aerobic exercise on asthma-related responses in adults J Allergy Clin Immunol 2011 127 2S AB223

[B44] Bonsignore MR La Grutta S Cibella F Effects of exercise training and montelukast in children with mild asthma Med Sci Sports Exerc 2008 40 3 405 412 10.1249/MSS.0b013e31815d9670 18379200

[B45] Bundgaard A Ingemann-Hansen T Schmidt A Halkjaer-Kristensen J Effect of physical training on peak oxygen consumption rate and exercise-induced asthma in adult asthmatics Scand J Clin Lab Invest 1982 42 1 9 13 10.3109/00365518209168043 7134793

[B46] Cochrane LM Clark CJ Benefits and problems of a physical training programme for asthmatic patients Thorax 1990 45 345 351 10.1136/thx.45.5.345 2116678 PMC462468

[B47] Fanelli A Cabral ALB Neder JA Exercise training on disease control and quality of life in asthmatic children Med Sci Sports Exerc 2007 39 9 1474 1480 17805077 10.1249/mss.0b013e3180d099ad

[B48] Fitch KD Blitvich JD MAR The effect of running training on exercise-induced asthma Ann Allergy 1986 57 90 94 3740562

[B49] Gunay O Onur E Yilmaz O Dundar PE Tikiz C Var A Effects of physical exercise on lung injury and oxidant stress in children with asthma Allergologia et Immunopathologia 2012 40 1 20 24 10.1016/j.aller.2010.10.006 21334801

[B50] Henriksen JM Toftegaard NT Effect of physical training on exercise-induced bronchoconstriction Acta Peadiatric Scan 1983 72 31 36 10.1111/j.1651-2227.1983.tb09659.x 6858683

[B51] Moreira A Delgado L Haahtela T Fonseca J Moreira P Lopes C Physical training does not increase allergic inflammation in asthmatic children Eur Respir J 2008 32 6 1570 1575 10.1183/09031936.00171707 18684843

[B52] Neder JA Nery LE AnC S Cabral ALB Fernandes ALG Short term effects of aerobic training in the clinical management of moderate to severe asthma in children Thorax 1999 54 3 202 206 10.1136/thx.54.3.202 10325894 PMC1745434

[B53] Onur E Kabaro A Ylu C GA¼nay O Var A Yilmaz O DA¼ndar P The beneficial effects of physical exercise on antioxidant status in asthmatic children Allergol Immunopathol 2011 39 2 90 95 10.1016/j.aller.2010.04.006 21242022

[B54] Sly R Harper R Rosselot I The effect of physical conditioning upon asthmatic children Ann Allergy 1972 30 86 94 4551436

[B55] Svenonius E Kautto R Arborelius JM Improvement after training of children with exercise-induced asthma Acta Paediatr Scan 1983 72 23 30 10.1111/j.1651-2227.1983.tb09658.x 6407276

[B56] Mendes FAR Almeida FM Cukier A Effects of aerobic training on airway inflammation in asthmatic patients Med Sci Sports Exerc 2011 43 2 197 203 10.1249/MSS.0b013e3181ed0ea3 20581719

[B57] Juvonen R Bloigu A Peitso A Silvennoinen-Kassinen S Saikku P Leinonen M Training improves physical fitness and decrease CRP also in asthmatic conscripts J Asthma 2008 45 237 242 10.1080/02770900701883790 18415833

[B58] Newcomb P Hunt A Rast P Cauble D Rowe N Li J Acute effects of walking environment and GSTM1 variants in children with asthma Biol Res Nurs 2012 14 1 55 64 10.1177/1099800410389167 21196426

[B59] Nickerson BG Bautista DB Namey MA Richards W Keens TG Distance running improves fitness in asthmatic children without pulmonary complications of changes in exercise-induced bronchospasm Pediatrics 1983 71 2 147 6823415

[B60] Silva PL Mello MT Cheik NC Sanches PL Correia FA Pian A Interdisciplinary therapy improves biomarkers profile and lung function in asthmatic obese adolescents Pediatr Pulmonol 2011 47 1 8 17 22170805 10.1002/ppul.21502

[B61] Segal RJ Reid RD Courneya KS Sigal RJ Kenny GP Prud'Homme DG Randomized controlled trial of resistance or aerobic exercise in men receiving radiation therapy for prostate cancer J Clin Oncol 2009 27 3 344 351 19064985 10.1200/JCO.2007.15.4963

[B62] Boule NG Haddad E Kenny GP Wells GA Sigal RJ Effects of exercise on glycemic control and body mass in type 2 diabetes mellitus: a meta-analysis of controlled clinical trials JAMA 2001 286 10 1218 1227 10.1001/jama.286.10.1218 11559268

[B63] Sigal RJ Kenny GP Wasserman DH Castaneda-Sceppa C White RD Physical activity/exercise and type 2 diabetes: a consensus statement from the American Diabetes Association Diabetes Care 2006 29 6 1433 1438 10.2337/dc06-9910 16732040

[B64] Heijink IH Van Oosterhout AJM Strategies for targeting T-cells in allergic diseases and asthma Pharmacol Ther 2006 112 2 489 500 10.1016/j.pharmthera.2006.05.005 16814862

[B65] Sood A Ford ES Camargo CA Jr Association between leptin and asthma in adults Thorax 2006 61 4 300 10.1136/thx.2004.031468 16540481 PMC2104595

[B66] Sutherland TJT Cowan JO Young S Goulding A Grant AM Williamson A The Association between Obesity and Asthma: interactions between systemic and airway inflammation Am J Respir Crit Care Med 2008 178 5 469 475 10.1164/rccm.200802-301OC 18565954

[B67] Mosen DM Schatz M Magid DJ Camargo J The relationship between obesity and asthma severity and control in adults J Allergy Clin Immunol 2008 122 3 507 511 10.1016/j.jaci.2008.06.024 18774387

[B68] Sutherland ER Obesity and asthma Immunol Allergy Clin North Am 2008 28 3 589 602 10.1016/j.iac.2008.03.003 18572109 PMC2504765

[B69] Beuther DA Sutherland ER Overweight, obesity, and incident asthma: a meta-analysis of prospective epidemiologic studies Am J Respir Crit Care Med 2007 175 7 661 666 10.1164/rccm.200611-1717OC 17234901 PMC1899288

[B70] Franklin PJ Stick SM The value of FeNO measurement in asthma management: the motion against FeNO to help manage childhood asthma–reality bites Paediatr Respir Rev 2008 9 2 122 126 10.1016/j.prrv.2007.12.004 18513672

[B71] Jartti T Wendelin-Saarenhovi M Heinonen I Hartiala J Vanto T Childhood asthma management guided by repeated FeNO measurements: a meta-analysis Paediatr Respir Rev 2012 13 3 178 183 10.1016/j.prrv.2011.11.002 22726875

[B72] Aaron SD Vandemheen KL Boulet LP McIvor RA FitzGerald JM Hernandez P Overdiagnosis of asthma in obese and nonobese adults Can Med Assoc J 2008 179 11 1121 1131 10.1503/cmaj.081332 19015563 PMC2582787

[B73] Pakhale S Doucette S Vandemheen K Boulet LP McIvor RA FitzGerald JM A comparison of obese and nonobese people with asthma: exploring an asthma-obesity interaction Chest 2010 137 6 1316 1323 10.1378/chest.09-2491 20154078

[B74] Pakhale S Sumner A Coyle D Vandemheen K Aaron S Correcting) misdiagnoses of asthma: a cost effectiveness analysis BMC Pulm Med 2011 11 27 10.1186/1471-2466-11-27 21605395 PMC3118954

[B75] Dogra S Kuk JL Baker J Jamnik V Exercise is associated with improved asthma control in adults Eur Respir J 2010 37 2 318 323 20530042 10.1183/09031936.00182209

[B76] Garcia-Aymerich J Varraso R Anto JM Camargo CA Jr Prospective study of physical activity and risk of asthma exacerbations in older women Am J Respir Crit Care Med 2009 179 11 999 1003 10.1164/rccm.200812-1929OC 19246716 PMC2689914

