# Supplementary material for: Effect of physical training on airway inflammation in bronchial asthma: a systematic review
Source: BMC Pulm Med. 2013 Jun 13;13:38. doi: 10.1186/1471-2466-13-38 (PMC3751945; doi:10.1186/1471-2466-13-38)
Supplement: Additional file 1 — Summary of included studies. This file provides details of the studies included in this systematic review including the number of subjects, mode of asthma diagnosis, asthma severity and control, current asthma pharmacotherapy, study design, training intervention (dose, duration, site, intensity and frequency of physical exercise), control intervention and outcome measures. [file 1471-2466-13-38-S1.pdf]

Additional File 1. Summary of included studies

Reference	No of subjects	Mean Age; Female %	Asthma Diagnosis, Asthma Severity, Medications,	Intervention	Study Design (control group)	Outcome Measures
Bonsignore MR et al. 2008	24	10 y; 20%	Positive MCT and skin test, Mild, controlled asthma; All patients on as needed SABA only	Aerobic circuit training 60 min, 2-4 times/week for 12 weeks at a moderate to vigorous intensity which gradually increased during the program.	RCT (Subjects randomized to placebo or LTRA, all exercised; we included the placebo arm only)	-EIB - defined as decrease by 10% of baseline FEV_1 at 10 or 20 minutes after maximal treadmill test – increased the slope by 2% until attainment of max Heart Rate or exhaustion, speed selected by subject)<br>-eNO (ppb)<br>-exhaled breath condensates of cysteinyl leukotrienes (po and nasal) (pg/mL)<br>-MCT (PC_{20})
Boyd, A. et al. 2011	9 (3 trained, 6 control) in progress	19-70y; ¥	Per ATS guidelines, evidence of reversibility via spirometry.<br>Mild to moderate persistent asthma; Medications not reported	Walking program 3 times/week over 12 weeks at 60-75% of age-predicted maximum heart rate	RCT	Interleukin-1 β<br>Pro-inflammatory targets in peripheral blood and nasal lavage
Bundgaard A, et al. 1982	27 (16 exercise, 11 control)	37 y; ¥	Decreased PEF >20% after running 6 min, and decreased FEV1>20% after 500 mcg of	15 min warm-up, heavy exercise: intervals and a circuit program for 45 min 2 times/week, for 8	RCT (control group: light exercise for	-change in PEFR pre and post-(1,3,5,10,15 min) 6 min run test (change = highest value pre- or post-

			histamine,<br>Moderate to severe asthma; 22% on ICS; 50% on oral steroids	weeks	60 min, 2 times/week x 8 weeks calisthenics, stretching, short run, power exercises heart rate< 120 bpm)	lowest post value) in mean +/- SEM L/min
Cochrane LM et al. 1990	36<br>(18 per group)	28 y; 61%	"Chronic mild to moderate asthma as defined by a need for regular prophylactic treatment and reproducible airway obstruction upon treatment withdrawal" , PC20 histamine all < 8 mg/mL,<br>Mild to moderate , controlled asthma; 58% on ICS; 5% on prednisone; 39% on cromoglycates	Warm-up, then 30 min varied aerobic exercises including cycling, jogging, aerobics, then a cool-down at 75% max. HR 30 min 3x/week for 12 weeks.	RCT (control: attendance at education sessions)	-Change in histamine PC_{20}
Emtner M et al. 1996	22***	41y; 77%	PEFR reversibility ≥ 20%, Mild to moderate asthma; 38% on low dose ICS alone; 58% on medium dose ICS±LABA; 4% on	Swimming for 45 min, 5 times/week plus education 5 days/week for 2 weeks at 80-90% maximum predicted heart rate and then 2	Prospective Cohort	-MCT (PC_{20})-exercise-induced asthma (defined by a fall in PEFR by > 15% (5, 10, 15, or 30 min after 6 min submaximal cycle ergometry test) units:

			medium dose ICS ±LABA and (oral steroid and/or oral β2 agonist and/or theophylline)	times/week for 8 weeks at Borg dyspnea 7-8 plus education 5 days/week		maximal decrease compared to initial value in L/min
Emtner M et al. 1998	57***	38 y; 66%	Diagnosis "per ATS criteria", Mild to moderate, controlled asthma;<br>12% on low dose ICS alone; 41% on medium dose ICS ±LABA;<br>45% on medium dose ICS ±LABA and (oral steroid and/or oral β2 agonist and/or theophylline)	Exercise on land, water, or water & land for 30 min at frequencies varying (0.2 to 3 times/week) for 156 weeks at varying intensities (from "light" Borg 4.1 to "heavy" Borg 6.6)	Prospective Cohort<br>3 year follow-up study post 10 weeks of rehabilitation (same subjects as in the two studies below with 3 year post-rehabilitation follow-up data)	-MCT (PC_{20})<br>EIB – defined as a drop in PEFR by 10% after a submaximal 6 min cycle ergometry test measured 0, 5, 10, 15, and 30 minutes post exercise (units: number of subjects meeting these criteria)
Emtner et al. 1998	31***	36y; 56%	Diagnosis "per ATS criteria", Mild to moderate, controlled asthma;<br>22% on low dose ICS alone; 41% on medium dose ICS ±LABA;<br>37% on medium dose ICS ±LABA and (oral steroid and/or oral β2 agonist and/or	Land or water training for 45 minutes 5 time/week for 2 weeks including 16 minutes of interval training at 80 – 100% maximum heart rate, training continued 2 times/week for the next 8 weeks at Borg dyspnea 7 - 8	RCT	-MCT (PC_{20})<br>-Exercise-induced asthma (% having a fall in PEFR >=10% after submaximal 6 min cycle ergometry measured 10 and 30 min after exercise). PEFR in L/min given in raw data form as well.

			theophylline)			
Engstom I, et al. 1991	6	11 y; 0%	No formal diagnostic criteria indicated but subjects from an Asthma-Allergy unit. Moderate to severe asthma; All on ICS	Swimming or gymnastics (goal HR 160-180) x 30 min (also a 15 min warm-up) 2 times/week, for 32 weeks. Accompanied by 15 min of relaxation & teaching at the end of every session	Prospective Cohort	-Bronchial reactivity to histamine (reaction dose mg/mL)
Fanelli A, et al. 2007	38 (21 exercise, 17 control)	11 y; 39%	Asthma per GINA guidelines,<br>Moderate to severe persistent, controlled asthma; All on moderate dose ICS	15 min warm-up, 15 min cool-down, 60 min of exercise (30 min aerobics, 30 min endurance exercises), 2 times/week for 16 weeks. Training intensity gradually increased over first 8 sessions, and heart rate goal (anaerobic threshold – respiratory compensation point) in incremental CPET. Intensity then gradually increased by 5% when child able to continuously perform the proposed activity for two consecutive days.	RCT (control: “non-exercising”)	-EIB (treadmill speed increased over < 4 min to reach 80% max HR & maintained x 6 min with spirometry pre- and 5, 10, 20 min post-exercise) with outcome being % decrease in FEV_1 of its pre-exercise value<br>-proportion of subjects with EIB+ (≥10% drop) or EIB+ severe (≥30% drop)
Fitch, KD	26	12 y; [¥]	1962 ATS definition of	15 min warm-up	RCT	-Maximum drop in PEFR

et all, 1986	(10 in the exercise group, 16 in the control group)		asthma diagnosis. Asthma severity unclear; Medications not reported	consisting of slow jogging and calisthenics, followed by running or games based on running (soccer, net ball, interval sprints, continuous running, volleyball) and circuit training with a target heart rate calculated as follows: 0.75 (Maximum heart rate – Resting heart rate) for 45 min, 4 times/week for 12 weeks.	(control: no alteration to normal lifestyle)	pre- and post treadmill exercise (speed and grade requiring 80% of VO_2 max)<br>Outcome measure: % of pre-exercise PEFR
Gunay, O. et al. 2012	30 (unclear how many in each group)	9.8y; 27%	Diagnosis of asthma based on history of recurrent cough and wheezing with prolonged expiration time which demonstrated clinical reversibility with short acting bronchodilator therapy, beta-2 agonist in subjects attending Paediatric Allergy and Pulmonology Department, Severity of asthma unclear; Medications not reported	Cycling at 80% of submaximal heart rate (submaximal heart rate being 50% higher than resting heart rate) plus 15 min warm-up 2x/week for 8 weeks and "pharmacologic therapy"	RCT	-Plasma matrix metalloproteinase -9 (MMP9), endothelin-1, MDA<br>-urine leukotriene E4
Henriksen	42	11 y; ¥	Decrease in PEF 15%	Warm-up 15 min, 60 min	RCT	-EIB (PEFR measured

JM, et al. 1983	(28 exercise, 14 control)		from baseline after submaximal exercise test, Mild to moderate asthma; Training group: 11% on theophylline, 11% on immunotherapy, 4% on prednisone; Control arm: less medications and only reliever therapy	of ball games, running, gymastics, circuit training & relay races 2x/week for 6 weeks	(control: usual physical activities in school and leisure time)	before exercise, after 2,4,5-6 min of exercise, and 3,5,10,15,20 post exercise with exercise being running on a treadmill for 5-6 min at 80-85% maximum workload): absolute fall from baseline in PEFR (baseline – lowest post-exercise/expected value x 100 expressed as a %), or % fall from baseline in FEV_1 (baseline – lowest post-exercise /baseline x 100 expressed as a %)
Juvonen R, et al. 2008	171	20 y; 0%	"Physician-diagnosed asthma" based on data from prior examination but not confirmed at the start of the study period. Physicians in Finland adhere to national guidelines when diagnosing asthma. Mild to moderate asthma; Medications not reported	Military service of duration 180, 270, or 362 days	Prospective Cohort	Serum highly sensitive CRP (change in mg/L) after the training program
Matsumoto I, et al. 1997	16 (8/group)	10 y; 12.5%	Diagnosis "per ATS criteria", Mild to moderate asthma; 50% on ICS, 75% on theophylline, 38% on	Swimming for 30 min (2 x 15 min intervals) 6 times/week for 6 weeks at 125% of lactate threshold.	RCT (control group: not specified)	-Histamine challenge test (PC_{20} mcg/mL)<br>-EIB: maximum % fall in FEV_1 after (5 & 15 min) swimming erogometer,

			cromoglycates			cycle ergometer at 100% and 175% lactate threshold (absolute and relative loads)
Mendes F. et al. 2011	51 (24 control, 27 trained)	37 y, 82%	Asthma diagnosis based on GINA guidelines. Moderate to severe persistent asthma; 100% on moderate to high dose budesonide, 94% on LABAs	Indoor treadmill for 30 min twice a week x 12 weeks initiated at 60% VO_2 max x 2 weeks, then increased to 70% or 80% VO_2max. If two consecutive asymptomatic sessions, increased HR by 5% up to a max of 80% max HR. Also underwent the same education & breathing exercises as control group	RCT (control: education and breathing exercises 30 min 2x/wek for 12 weeks)	-Induced sputum: total cell count ($x10^6$), eosinophils, PMNs, macrophages (%)<br>-FeNO (ppb)
Moreira, A et al. 2008	32 (16 per group but measurements done on 14 – 16 depending on which outcome)	13 y; 59%	Method of asthma diagnosis unknown but “atopic” and “asthmatic” and followed in a University clinic.<br>Mild, controlled asthma; All on low to moderate dose ICS<br>65% on LABA<br>26% on LTRA	Mixed submaximal training (aerobic, strength, balance) for 30-35 min with a total of 17-20 min of warm-up/cool-down 2 times/week for 12 weeks	RCT (control: continued their usual routine)	-eNO (ppb)<br>-serum: eosinophils (%), highly sensitive CRP (mg/dL), total IgE (kUA/L), mite-specific IgE (kUA/L), ECP (mcg/L)<br>- MCT (PD_{20}, measured in mg)
Neder JA, et al. 1999	42 (26 in exercise,	12.4 y; 43%	Method of diagnosis unclear but referred from	10-15 min warm-up of calisthenics, stretching,	RCT (control	-EIB: spirometry measured 5, 10, 20 min post-exercise

	16 control) BUT EIB testing done in only those with normal baseline FEV1 (n = 35)		an asthma management centre,<br>Moderate to severe asthma; All on ICS, 21% on prednisone	then 30 min cycling with target heart rate set to anaerobic threshold and increased q 2 weeks to an intensity the subject could endure for 30 min, 3 time/week for 8 weeks	group: "untrained")	(cycle ergometer with workload increased at 1 min to 80% predicted HR and then maintained for 6 minutes)<br>(outcome: % of participants considered to have EIB – present if FEV_1 decreased by ≥10% of pre-exercise value)
Newcomb, P. et al. 2012	24	5-12 y; 62.5%	Physician diagnosed asthma from primary care clinics, ER, short-stay hospital, Mild asthma;<br>Medications not reported	Walking program at a brisk pace for 30 min 5x/week for 2 weeks	Cohort	-FeNO
Nickerson BG et al., 1983	15	11 y; 20%	ATS criteria of asthma diagnosis, Severe asthma;<br>All on ICS and theophylline, 13% on prednisone; 60% on oral sympathomimetics	Running (based on distance of 3.2 km so not timed, but subjects encouraged to compete with each other & with their previous times), 4 times/week for 6 weeks	Prospective Cohort	-EIB 5 and 10 min post-exercise (measured by change in airway resistance, fall in FEV_1, or fall in FEF 25-75% after a graded maximal exercise stress test on a bicycle)<br>(outcome was % baseline for all three parameters pre and post training program)
Onur E, et al. 2011	30 (15/group)	10 y; 27%	Clinical history and clinical reversibility with short-acting B2 agonist, Mild asthma;	Cycling for 45 min at 80% of submaximal heart rate (submaximal HR being 50% higher than	RCT Control group received	-Plasma malondialdehyde (MDA), glutathione peroxidase (GSH-PX), superoxide dismutase

			None on ICS	resting HR) plus 15 min warm-up 2x/week for 8 weeks plus fluticasone 250 mcg/day	only fluticasone in the same dose as the intervention group	(SOD), total nitric oxide (NO)
Silva PL, et al. 2011	26	16 y; 75%	ATS guidelines<br>Clinical asthma & reversibility post-bronchodilator.<br>Mild to moderate asthma; Medications not reported	Aerobic training 30 min and resistance training 30 min 3 times/week x 52 weeks<br>(running on treadmill at cardiac frequency of ventilator threshold)<br>-also got psychological & nutritional therapy 1x/wk	Prospective Cohort	-Serum CRP (ng/mL), leptin (ng/mL), adiponectin (ng/mL), Il-6 described in the abstract presented at the 11th International Congress on Obesity in 2010, but not in the results section of the published article in 2011<br>-serum eosinophils
Sly RM, et al. 1972	26 (13 in each group) but only 11 asthmatics completed the EIB test	9-13 y; ¥	All had recurrent episodes of airway obstruction remitting spontaneously or following bronchodilator treatment, a history of perennial asthma symptoms, and were attending a pediatric allergy clinic. Moderate to severe asthma; Medications not reported	Swimming, calisthenics, tumbling, work-out on bars, rope climbing, running, relay races 120min, 3 times/week for 12 weeks and a schedule of exercises to be done at home (relaxation, breathing, strengthening). Also participated in breathing exercises	RCT (control: not specified)	-PEFR immediately pre and 10 min post treadmill exercise (8 minutes, 10º grade, 3 miles/hour)<br>Outcome: mean decrease in PEFR following exercise before and after the program
Svenonius E, et al.	50 (40 in the exercise	"Children" age not reported;	History of asthma on exertion and allergen-	Interval training, running, swimming with a goal	RCT (control	- FEV_1 pre- and post (4 min +/- 1 min) - treadmill test

1983	group, 10 in the control group)	¥	mediated asthma, volume of trapped gas increased by ≥ 24 mL after treadmill exercise to HR 170 bpm. Mild Asthma; None on anti-inflammatory treatment	heart rate of 170 bpm for 30 min (preceded by 5 min warm-up), 2 times/week, for 12 – 16 weeks (15 participants in the training group did voluntary activity but commonly did interval training)	group did not change their lifestyle)	(6 min, 5% grade, speed increased to meet a HR of 170 bpm)<br>Outcome: mean differences in % of predicted normal values before and after training

¥Sex distribution unclear
SABA – Short Acting Beta-2 Agonist
ICS – Inhaled corticosteroids
LABAs- Long Acting Bronchodilators
LTRA – Long Acting Leukotrian Antagonists
RCT – Randomized Controlled Trial
MCT – Methacholine Challenge Test
EIB – Exercise Induced Bronchoconstriction
PEF/R – Peak Expiratory Flow/Rate
FeNO- Fractional excretion of Nitric Oxide
CPET – Cardio Pulmonary Exercise Test
ATS –American Thoracic Society
GINA- Global Initiative for Asthma
